# Supplementary material for: Lactic Acid Bacteria (LAB) and Their Bacteriocins for Applications in Food Safety Against Listeria monocytogenes
Source: Antibiotics (Basel). 2025 Jun 3;14(6):572. doi: 10.3390/antibiotics14060572 (PMC12190153; doi:10.3390/antibiotics14060572)
Supplement: Supplementary file 1 [file antibiotics-14-00572-s001.zip › antibiotics-3667002-supplementary.pdf]

Supplementary material submitted along with the manuscript:

Article

Lactic Acid Bacteria (LAB) and Their Bacteriocins for Applications in Food Safety Against *Listeria monocytogenes*

Cristian Piras [1,†], Alessio Soggiu [2,†], Viviana Greco [3,4], Pierluigi Aldo Di Ciccio [5], Luigi Bonizzi [2], Anna Caterina Procopio [1], Andrea Urbani [3,4] and Paola Roncada [1,*]

[1] Department of Health Sciences, Magna Græcia University of Catanzaro, 88100 Catanzaro, Italy; c.piras@unicz.it (C.P.); annacaterina.procopio@unicz.it (A.C.P.)
[2] One Health Unit, Department of Biomedical, Surgical and Dental Sciences, University of Milan, Via Pascal 36, 20133 Milan, Italy; alessio.soggiu@unimi.it (A.S.); luigi.bonizzi@unimi.it (L.B.)
[3] Department of Basic Biotechnological Sciences, Intensivological and Perioperative Clinics, Università Cattolica del Sacro Cuore, 00168 Rome, Italy; viviana.greco@unicatt.it (V.G.); andrea.urbani@unicatt.it (A.U.)
[4] Department Unity of Chemistry, Biochemistry and Clinical Molecular Biology, Department of Diagnostic and Laboratory Medicine, Fondazione Policlinico Universitario A. Gemelli IRCCS, 00168 Rome, Italy
[5] Department of Veterinary Sciences, University of Turin, Largo Braccini 2, Grugliasco, 10095 Torino, Italy; pierluigialdo.diciccio@unito.it
[*] Correspondence: roncada@unicz.it
[†] The authors contributed equally to this work.

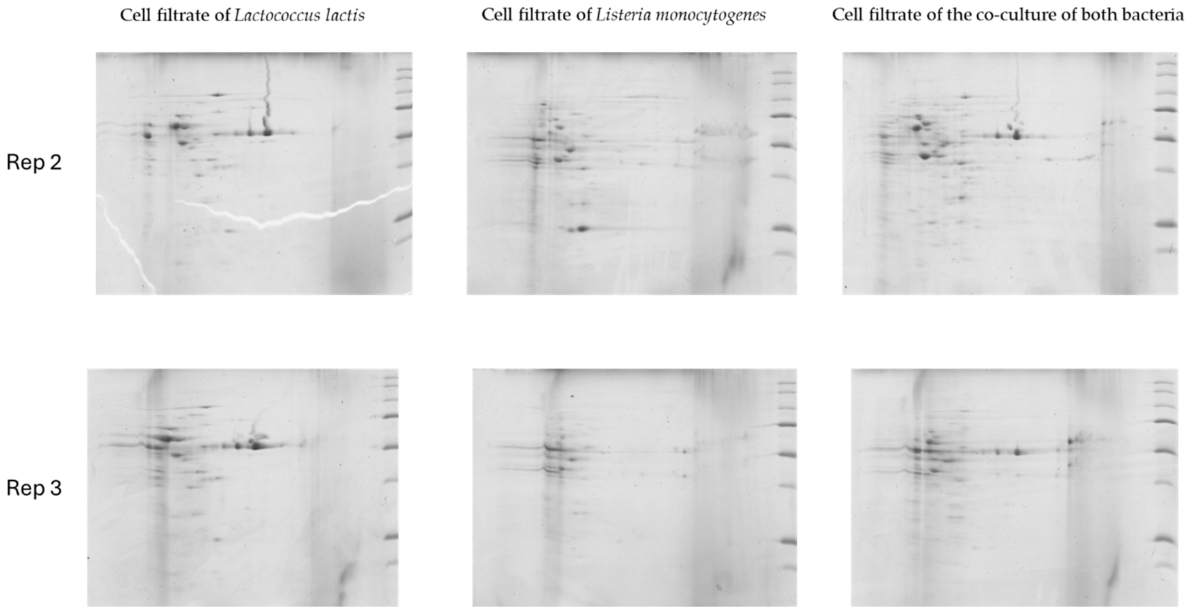

Figure S1. Experimental replicates number 2 and 3 of the 2D-electrophoresis experiments.

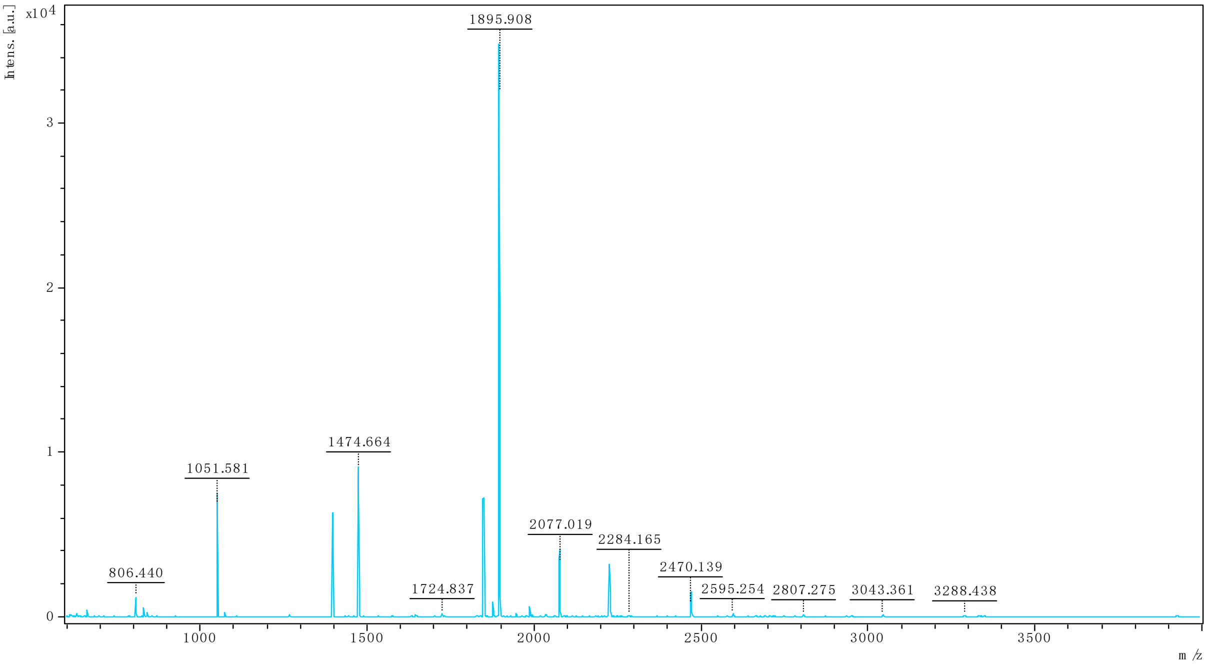

Figure S2. MALDI-TOF spectrum obtained from the excision and the trypsin digestion of the protein spot indicated by the arrow in figure 1.

Table S1. Shotgun MS analysis table listing the proteins of *Lactococcus lactis* and *Listeria monocytogenes* in single culture.

Bacterial culture	Uniprot acession number	Protein description	Calculated Femtomoles	Femtomoles SD	Nanograms	Nanograms SD
LL	P13068	Lantibiotic nisin A OS Lactococcus lactis subsp lactis GN spaN PE 1 SV 1	52,00	3,46	2,07	0,37
LL	P22865	Secreted 45 kDa protein OS Lactococcus lactis subsp cremoris strain MG1363 GN usp45 PE 1 SV 3	nq		nq	
LL	Q9CG42	50S ribosomal protein L7 L12 OS Lactococcus lactis subsp lactis strain IL1403 GN rplL PE 3 SV 1	10,79	2,68	0,13	0,03
LL	Q9CI64	DNA binding protein HU OS Lactococcus lactis subsp lactis strain IL1403 GN hup PE 3 SV 1	nq	nq	nq	
LM	C1KVG6	Aspartate tRNA ligase OS Listeria monocytogenes serotype 4b strain CLIP80459 GN aspS PE 3 SV 1	7,17	0,15	0,48	0,01
LM	C1KY94	Enolase OS Listeria monocytogenes serotype 4b strain CLIP80459 GN eno PE 3 SV 1	71,20	1,97	3,19	0,16
LM	P21171	Probable endopeptidase p60 OS Listeria monocytogenes serovar 1 2a strain ATCC BAA 679 EGD e GN i	226,36	24,61	5,31	0,58
LM	Q71WB8	Elongation factor G Listeria monocytogenes serotype 4b (strain F2365)	nq	nq	nq	nq
LM	Q71XG7	Tryptophan--tRNA ligase Listeria monocytogenes serotype 4b strain F2365	nq	nq	nq	nq
LM	Q720K2	Uracil DNA glycosylase 2 OS Listeria monocytogenes serotype 4b strain F2365 GN ung2 PE 3 SV 1	22,07	5,84	0,57	0,15
LM	Q8Y9X7	Uracil DNA glycosylase 1 OS Listeria monocytogenes serovar 1 2a strain ATCC BAA 679 EGD e GN ung	12,35	1,13	0,31	0,03
LM	Q8YAA3	50S ribosomal protein L7 L12 OS Listeria monocytogenes serovar 1 2a strain ATCC BAA 679 EGD e GN	375,90	43,03	4,76	0,60

Table S2. Shotgun MS analysis table listing the proteins of *Lactococcus lactis* identified during bacterial competition.

Competition LM+LL	Uniprot acession number	Protein description	Calculated Femtomoles	Femtomoles SD	Nanograms	Nanograms SD
LL	D2BNK8	Uncharacterized protein OS Lactococcus lactis subsp lactis strain KF147 GN usp PE 4 SV 1	nq		NQ	nq
LL	F2HKA4	Nisin NisinA OS Lactococcus lactis subsp lactis strain CV56 GN nisA PE 4 SV 1	nq		nq	nq
LL	H5SXJ3	GTNG 0265 lantibiotic antimicrobial peptinisin Ade OS Lactococcus lactis subsp lactis IO 1 GN nisA	nq		nq	

LL	P22865	Secreted 45 kDa protein OS Lactococcus lactis subsp cremoris strain MG1363 GN usp45 PE 1 SV 3	21,70	0,63	116,00	3,46
LL	P29559	Lantibiotic nisin Z OS Lactococcus lactis subsp lactis GN nisZ PE 1 SV 1	669,03	86,32	3,97	0,51
LL	Q45RP0	Lantibiotic nisin A Fragment OS Lactococcus lactis subsp lactis GN nis PE 4 SV 1	nq		nq	
LL	Q7DH25	Nisin Z OS Lactococcus lactis subsp lactis GN nisZ PE 4 SV 1	nq		nq	
LL	Q9CDJ1	Putative uncharacterized protein usp45 OS Lactococcus lactis subsp lactis strain IL1403 GN usp45	nq		nq	

Table S3. Shotgun MS analysis table listing the proteins of *Listeria monocytogenes* identified during bacterial competition.

Competition LM+LL	Uniprot acession number	Protein description	Calculated Femtomoles	Femtomoles SD	Nanograms	Nanograms SD
LM	B8DHE7	Septation ring formation regulator EzrA OS Listeria monocytogenes serotype 4a strain HCC23 GN ezrA	10,88	1,35	0,73	0,09
LM	C1KY63	Glycine cleavage system H protein OS Listeria monocytogenes serotype 4b strain CLIP80459 GN gcvH P	93,87	3,18	1,29	0,05
LM	C1KY94	Enolase OS Listeria monocytogenes serotype 4b strain CLIP80459 GN eno PE 3 SV 1	192,93	14,09	8,97	0,65
LM	C1KYI0	50S ribosomal protein L11 OS Listeria monocytogenes serotype 4b strain CLIP80459 GN rplK PE 3 SV 1	168,99	37,51	2,48	0,53
LM	C1KYI3	50S ribosomal protein L7 L12 OS Listeria monocytogenes serotype 4b strain CLIP80459 GN rplL PE 3 S	404,85	3,26	5,06	0,04
LM	C1L2V9	DNA mismatch repair protein MutS OS Listeria monocytogenes serotype 4b strain CLIP80459 GN mutS PE	36,41	0,00	3,57	0,00
LM	G2JZ74	D alanine aminotransferase OS Listeria monocytogenes serotype 1 2a strain 10403S GN dat PE 3 SV 1	14,96	0,92	0,49	0,03
LM	Q71X61	Glucose 6 phosphate isomerase OS Listeria monocytogenes serotype 4b strain F2365 GN pgi PE 3 SV 1	29,57	2,22	1,47	0,11
LM	Q8Y4L2	Glycine cleavage system H protein OS Listeria monocytogenes serovar 1 2a strain ATCC BAA 679 EGD	nq		nq	
LM	P21171	Probable endopeptidase p60 OS Listeria monocytogenes serovar 1 2a strain ATCC BAA 679 EGD e GN i	nq		nq	
LM	Q71WB8	Elongation factor G Listeria monocytogenes serotype 4b (strain F2365)	nq		nq	
LM	Q71XG7	Tryptophan--tRNA ligase Listeria monocytogenes serotype 4b strain F2365	nq		nq	
LM	Q8Y9X7	Uracil DNA glycosylase 1 OS Listeria monocytogenes serovar 1 2a strain ATCC BAA 679 EGD e GN ung	9,96	0,56	0,25	0,01

LM	Q8YAA3	50S ribosomal protein L7 L12 OS Listeria monocytogenes serovar 1 2a strain ATCC BAA 679 EGD e GN	300,74	18,56	3,82	0,22

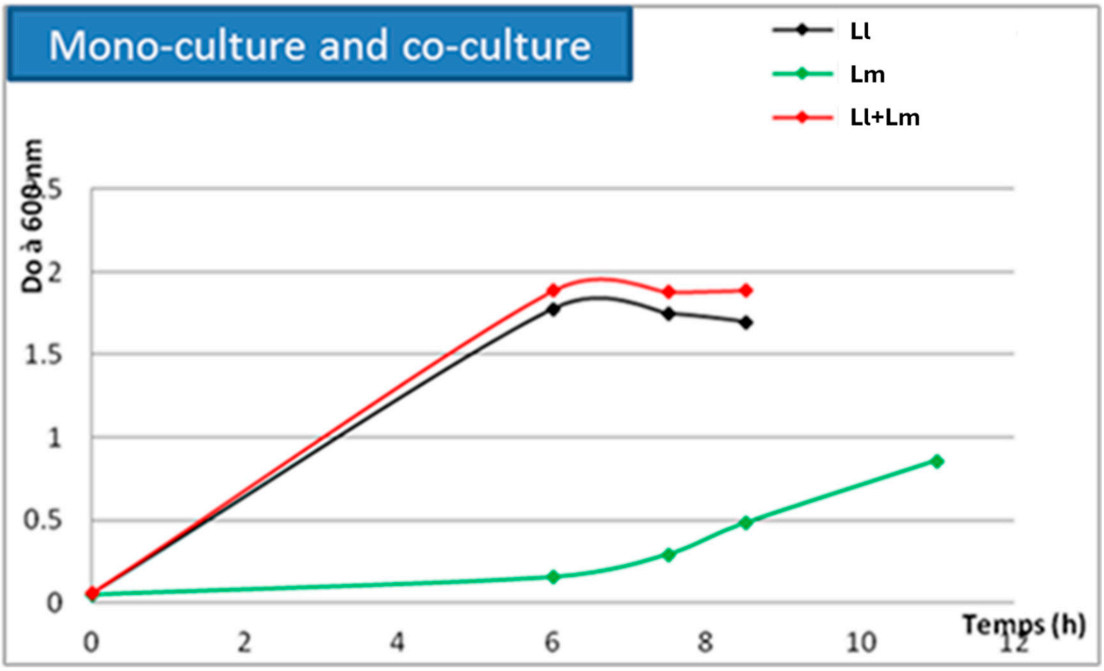

Figure S3. Growth curves of *Listeria monocytogenes (Lm)* and *Lactococcus lactis (Ll)* in monoculture and in co-culture.
